# An AI-based intervention for improving undergraduate STEM learning

**DOI:** 10.1371/journal.pone.0288844

**Published:** 2023-07-19

**Authors:** Mohammad Rashedul Hasan, Bilal Khan

**Affiliations:** 1 Electrical and Computer Engineering, University of Nebraska-Lincoln, Lincoln, NE, United States of America; 2 Depts. of Community & Population Health, and Computer Science & Engineering, Lehigh University, Bethlehem, PA, United States of America; Carnegie Mellon University, UNITED STATES

## Abstract

We present results from a small-scale randomized controlled trial that evaluates the impact of just-in-time interventions on the academic outcomes of N = 65 undergraduate students in a STEM course. Intervention messaging content was based on machine learning forecasting models of data collected from 537 students in the same course over the preceding 3 years. Trial results show that the intervention produced a statistically significant increase in the proportion of students that achieved a passing grade. The outcomes point to the potential and promise of just-in-time interventions for STEM learning and the need for larger fully-powered randomized controlled trials.

## Introduction

Even as the number of jobs requiring science, technology, engineering, and mathematics (STEM) knowledge and skills rapidly increase [1], retention rates in post-secondary STEM fields have fallen below 50% [2]. The graduation rate of STEM students is roughly 20% below their counterparts in non-STEM majors [3]. This is attributed to students’ poor academic performance, particularly in the first few years of college [4]. Improving student performance in STEM programs is thus a critical national need. Many large-scale systemic reforms might help, including department-wide implementation of evidence-based practices, faculty development, and leadership, adoption of successful curricular approaches, stronger teacher preparation programs, student learning communities, professional development of faculty, etc. [2, 5, 6]. Yet, the rate at which systemic changes can take place is limited by each institution’s financial resources and inertia [7], so it is important to develop alternative, cost-effective, incremental, and contextually appropriate interventions that might improve STEM performance. We report on one such attempt here.

Education researchers approach this need for intervention from many angles, e.g., designing active learning strategies to improve learning in the classroom [8], developing light-touch interventions to improve learning outside the classroom [9], and forming STEM learning communities to address the social aspects of learning [10, 11], etc. Other approaches engage psychological interventions such as online sessions conveying a growth mindset [12], performance-related warnings through course management system-based alerts [13], and emails containing grade forecast [7] to improve academic achievement. Such approaches implicitly leverage non-cognitive psychological drivers (e.g., motivation) of academic performance [6], drawing support from Social Cognitive Theory [14].

Most recently, Artificial Intelligence (AI)-based strategies have been proposed for the delivery of psychological interventions [13, 15]. These approaches involve the creation of predictive models that use students’ recent performance data (e.g., academic scores at the beginning of the semester) to forecast their final course outcomes. Such forecast messages are seen as interventions that serve to both inform students and motivate them to improve their academic performance [7, 13, 16]. The customizability and low implementation costs of AI-based solutions make them a potentially cost-effective, scalable approach for improving academic achievement, particularly in courses during the first two years of college, where STEM curriculum is fairly standardized, and performance is critical to long-term student retention [13, 16, 17].

This article presents an ML-based method for providing just-in-time interventions among undergraduate STEM students and evaluates its efficacy via a small randomized control trial.

## Materials and methods

### Study cohort

The study cohort consisted of 65 students who enrolled in the introductory course on discrete structures in the Fall of 2019 at the University of Nebraska-Lincoln (UNL). The course had prerequisites of introductory programming and precalculus-level mathematics. All 65 of the students were first-year students who were enrolled in the study by signing a written informed consent form. The study was approved by the UNL’s Institutional Review Board (IRB #: 20180118001EX).

All students were informed at the beginning of class that they would be receiving 3 messages from “an AI” at a regular interval and that these 3 messages would contain “a forecast of your future performance”. More specifically, the AI would determine if their prospects were “Good”, “Fair”, “Prone-to-Risk”, “At-Risk”, but in some cases, the AI might declare that it was “Unable to make a prediction”. Students were told that the messages they received would correspond to the final grades the AI had predicted for them, in accordance with Table 1.

**Table 1:** Mapping of the AI’s forecast to the AI’s message.

AI’s forecast of final grade	Message from AI
90% ⩽ grade ⩽ 100%	“You are Good”
80% ⩽ grade < 90%	“You are OK”
70% ⩽ grade < 80%	“You are Prone-to-Risk”
0% ⩽ grade < 70%	“You are At-Risk”

The AI was instrumented by an ML app accessible through computer/cellphone browsers. Students were instructed to use their course management system (i.e., Canvas) credentials to log in to the app. When new forecasting was computed by the AI, the app notified the students by sending an automatic message to their email accounts. The message contained their forecasted performance. Students could also check their forecasting messages by logging in to the app. The app does not provide any other information apart from three periodic forecasting messages.

### Randomized assignment

Just prior to week 6, the cohort was split into two groups. A randomly chosen one-half (33) of the students were assigned to the control group, and the remaining (32) were assigned to the intervention group. Students were not informed about the fact that a randomized assignment had taken place, or which group they were placed in.

All students (regardless of group) received 3 messages over the course of the semester, at the end of weeks *t* = 6, 9, and 12. The message at week 6 preceded the course midterm exam by 7 days; the message at week 12 preceded the final exam by 7 days; the message at week 9 was transmitted 1/3 of the way through the time interval between the midterm and final exams. These 3 timepoints were chosen in advance by contemplating natural pivots in the course delivery.

### Intervention group

Within the intervention group, each student received a message at the end of week *t* = 6, 9, and 12. Each message indicated whether the AI had determined the student’s prospects to be “Good”, “Fair”, “Prone-to-Risk”, or “At-Risk”. To generate the message for each student, data on the student’s formal assessments (to date) were fed as input into an appropriate previously trained predictive ML model. The model’s prediction was then translated into a message in accordance with Table 1. The AI-generated messages were delivered to each student via the app.

### Control group

Within the control group, each student received a message at the end of week *t* = 6, 9, and 12. Each message stated that the AI had been “Unable to make a prediction” concerning the student’s prospects in the course. These messages were delivered to each student via the app.

### Predictive machine learning models

The predictive models described in this section were developed by the authors in previous work [15], and only a brief summary description is provided here. Three distinct predictive machine learning models were developed, $M_{6}$, $M_{9}$, and $M_{12}$, to be used in determining intervention message content at weeks 6, 9, and 12, respectively.

The **training data set** consisted of academic assessments collected from 537 students who were enrolled in the same course between Fall 2015 and Spring 2018. The number of cases in the training data set was thus 537. The dimensions of the data set were 17, consisting of 17 numerical predictors (numerical scores on homework assignments, quizzes, and exams), along with each student’s numerical final grade. The numerical grade was replaced with a categorical label of “A”, “B”, “C” or “Below C” using the binning scheme described in Table 2.

**Table 2:** Binning of numerical final grade.

Numerical final grade	Label
90% ⩽ grade ⩽ 100%	“A”
80% ⩽ grade < 90%	“B”
70% ⩽ grade < 80%	“C”
0% ⩽ grade < 70%	“Below C”

Before building the model, **feature selection** was carried out by retaining only those predictors whose Pearson correlation with the final grade exceeded 0.45. The resulting features selected are presented in Table 3.

**Table 3:** Outputs of the feature selection process.

Model	Data Considered	Features selected
$M_{6}$	Weeks 1–6	Quiz 1–3 & Homework 1, 2
$M_{9}$	Weeks 1–9	Quiz 1–5 & Homework 1, 2, 3 & Midterm 1
$M_{12}$	Weeks 1–12	Quiz 1–7 & Homework 1, 2, 3, 4 & Midterm 1

Rather than training a single classifier to predict all 4 class labels (“A”, “B”, “C”, and “Below C”) in one shot, we followed a hybrid approach [18]. Building each model $M_{t}$ (*t* = 6, 9, 12) required the training of two classifiers:

$C_{t}^{1}$ is a 3-label classifier that predicts either “A”, “B” or “C and Below”. It is trained on a transformed data set where the students who were labeled “C” or “Below C” have been relabelled as “C and Below”. The distribution of the labels for this classifier is: A = 252, B = 156, and C and Below = 129.

$C_{t}^{2}$ is a binary classifier that predicts either “Below C” or “not Below C”. It is trained on a transformed data set where the students who were labeled “A”, “B”, or “C” have been relabelled as “not Below C”. The distribution of the labels for this classifier is: not Below C = 396 and below C = 141.

The predictions of the two classifiers $C_{t}^{1}$ and $C_{t}^{2}$ are combined to create the predictive model $M_{t}$ as follows:

- If $C_{t}^{1}$ predicts “A” or “B” then this output is taken to be the output of $M_{t}$.
- If $C_{t}^{1}$ predicts “C and Below” then $C_{t}^{2}$ is consulted:
  - If $C_{t}^{2}$ predicts “Below C” then that is taken to be the output of $M_{t}$.
  - If $C_{t}^{2}$ predicts “not Below C” then the output of $M_{t}$ is “C”.

This 2-stage design was chosen to address challenges associated with a lack of data and features, especially in early predictions (e.g., the $M_{6}$ model). Performance measures for the $M_{6}$, $M_{9}$, and $M_{12}$ models were described by the authors in their prior work [15], but are summarized in Table 5 in S1 Appendix.

### Measures of impact on student outcomes

We are interested in assessing the impact of the intervention on student performance outcomes. Specifically, we would like to know whether the intervention significantly increased the proportion of students who passed the course (i.e., achieved a grade > 64). To answer this question, we considered the 2x2 contingency table containing counts, where the groups (columns) were taken as Intervention and Control, while the outcomes (rows) were taken to be Failed (<= 64) or Passed (> 64). By design, the total number of individuals in each of the intervention versus control groups was fixed, so the columns of the contingency table were conditioned, while the row sums were not. Our assignment of individuals to intervention versus control was at random; this, together with our assumption that social network effects were minimal, allowed us to conclude that responses aggregated in the table were indeed independent. Given these data characteristics, together with the small sample size, we chose to apply Barnard’s test [19]– an unconditional exact test for two independent binomials. Let *p*_*I*_ denote the binomial probability of “Failing” for members of the intervention group and *p*_*C*_ the analogous probability for members of the control group. Our null hypothesis is then *H*_0_: *p*_*I*_ ≥ *p*_*C*_ and alternative hypothesis *H*_1_: *p*_*I*_ < *p*_*C*_. The hypothesis was tested at the 5% significance level via a one-sided Barnard’s test engaging the Wald statistic with unpooled variance, by using the implementation in the scipy library [20] (see “Supporting information” section for code). The findings from this analysis are presented in the Results section below.

## Results

Prior to the first intervention at week 6, we did not observe a significant difference in the distribution of various performance types among the control and treatment groups.

### Impact on student outcomes

Table 4 shows the 2x2 contingency table derived from the data of our randomized control trial.

**Table 4:** Contingency table.

	Intervention	Control	Totals
Failed	3	9	12
Passed	29	24	53
Totals	32	33	65

Barnard’s test yields 1.92 as the value of the Wald statistic, with a p-value of 0.0352. It follows that under the null hypothesis (that the intervention does not lower the chance of a student failing), the probability of obtaining trial results at least as extreme as the observed data is approximately 3.5%. Since this p-value is less than our chosen significance level of 5%, we have sufficient evidence to reject the null hypothesis in favor of the alternative.

Additionally, Relative Risk (RR) was used to assess the substantive significance within the Randomized Controlled Trial (RCT). The RR was found to be 0.34. Given that the RR value was <1, the intervention was confirmed to reduce the number of students below the threshold relative to the control group.

### Acceptability of the intervention

We conducted a general user survey on the preliminary version of the AI-based app at the end of the Fall 2019 semester. The survey was voluntary and there was no incentive provided for completing it (e.g., no financial incentives or extra credit points). A total of 40 (of the 65) students completed the survey, of which 8 were female and 32 were male. Of the 40 respondents, 24 students belong to the intervention group, while 16 were in the control group. The survey gathered data on students’ experience and demographics through a series of multiple-choice questions, the results of which are presented below:

Question Q1 read *Did you receive the message “Unable to make a prediction” during the semester?* The question was used to identify membership in the intervention arm since only the students in the control arm received this message.

Question Q2 read *How many times have you used the performance forecasting app during the semester?* This question was used to identify “active users” within the intervention arm. Survey data showed that 14 students (58% of the 24 who received the intervention) actively used the app (“Few times a month” = 7, “Every 2/3 weeks” = 5, and “Almost every week after the prediction was made” = 2). The other 10 students “never” used the app. Further analysis showed variability in usage by gender: Of the 24 students in the intervention arm, 6 were female and 18 were male. However, among the females, only 17% (1 of 6) were active users (5 female students “never” used the app, and the remaining 1 female student used the app “Few times a month”), while among the males 78% (14 of 18) were active users.

Question Q3 read How useful were the predictions? There were 4 possible answers: “Very useful”, “Somewhat useful”, “Not at all useful”, and “I have never looked at the predictions”. Data from this question revealed that 12 of the 14 active users (86%) reported finding the app’s predictions to be useful (“Very useful” = 5, “Somewhat useful” = 7, and “Not at all useful” = 2).

Question Q4 read Did you put more effort into your studies after seeing the predictions? There were 4 possible answers: “Yes”, “Not always”, “No”, and “I have never looked at the predictions”. The question was used to determine that 12 of the 14 active users (86%) reported they believed they put more effort in after seeing the predictions (“Yes” = 6, “Not always” = 6, and “No” = 2).

### Discussion

Though we observed an overall positive impact of the AI-based interventions on students’ academic outcomes, we identified some limitations in the design of the interventions, analysis of the study results, and acceptability survey of the interventions.

#### Unintended intervention to the control group

Our app attempted to mask the randomized assignment into the control/intervention arms by sending the control group students messages that read “Unable to make a prediction” whenever members of the intervention group received a forecast-based prompt. These “deceptive” messages might have influenced the outcome of some students in the control group and impacted their course-related behaviors (compared to if they had received no messages at all). Unfortunately, in the present study, we did not collect any information about the psychological impact of these deceptive messages on students in the control group. We acknowledge that there are potential ethical concerns surrounding deception in our experiments, given the unknown impacts. Our future studies will attempt to circumvent the need for deceptive messages altogether or will, at minimum, seek to measure the impact of deceptive messages to ascertain that no harm is incurred.

#### Limited to intent-to-treat analysis

Our app did not capture metadata on user engagement with the app, so we were not able to evaluate treatment compliance except via self-report at the end-of-study survey. Unfortunately, in hindsight, the granularity of this usage/acceptability survey was too coarse to enable refined analyses. For example, in question 2, we intended to identify “active users” in the intervention arm based on whether they reported using the app “At least a few times in a month”. However, since the app sent only three messages over the semester, this question may have yielded an undercount of active users. We plan to alleviate these limitations in our future study by sending intervention messages more frequently and collecting metadata about participant viewing behaviors to support more nuanced as-treated analysis.

#### Limitations to generalizability

Our system is based on the premise that a model trained on previous offerings of a course can subsequently be used to deliver interventions to students in future offerings of the same course. This requires a clear correspondence between assessment items (i.e., homework, quizzes, exams) across semesters. In our RCT, the same instructor taught the same curriculum across all semesters from which the model was trained, and the intervention deployed. More broadly, if each assessment’s topics remain the same, but the questions change, we believe it may be possible to limit the impact of cross-semester heterogeneity through data preprocessing and normalization. If, however, the topics are fundamentally altered over time, it will be challenging to train and apply a model. An ML-based intervention system that relies on academic assessment data alone may not be effective if the assessment topics vary significantly over time. It would be interesting to investigate if the inclusion of non-academic data (e.g., course-related behavior, science identity, socioeconomic background, social connectedness, etc.) makes the interventions more robust to variability in the assessment instruments over time. In the future, we would like to explore such questions by acquiring non-academic features and using them alongside assessment data in the next version of our ML system.

#### Limitations due to scale

Our research findings are limited by statistical power implications of the small cohort size (65 students), short duration (one semester), predictor granularity (17 timepoints), and intervention frequency (3 timepoints). A future RCT that is larger on any/all of these axes will allow us to test richer hypotheses, such as whether it is possible to cluster students based on their distal characteristics and proximal trajectories, towards the design of tailored interventions.

## Conclusions

We conducted a small-scale randomized controlled trial to evaluate the effectiveness of AI-based interventions in improving undergraduate STEM education. The study shows the potential for leveraging AI to build this type of intervention system:

- We found that the messaging intervention increased the proportion of students who passed (i.e., achieved a final grade > 64) and that this increase was statistically significant (*p* = 0.0352).
- Exit surveys from the RCT found that a significant fraction (∼58%) of students had used the app frequently, and of those, a significant fraction (∼86%) felt that messages were helpful and that they worked harder after receiving them. Male students were ∼4 times more likely than females to use the app (72% versus 17%).

## Future work

In the short term, we plan to address the limitations presented in the Discussion section by collecting data from larger, more carefully designed RCT. In addition to this, we plan to perform robustness testing. Longer term, we will pursue the promises of AI to tailor interventions by reflecting the natural heterogeneity among students. We will approach this by computing a typology of students based on their responsiveness to messaging interventions drawn from a range of design parameters. While other researchers have built classification schemes based on students’ assessment scores and cognitive factors [21–23], few have considered students’ relative responsiveness to interventions as the basis of a typology. Given such a typology, an AI system that can rapidly infer each student’s type might be able to generate intervention messaging with tone and content optimized to yield favorable outcomes. This line of inquiry would be a natural extension of prior efforts in machine learning that have sought to automate the efficient determination and updating of student classifications [18, 24–26], and allow for tailored interventions throughout the semester.

## Supporting information

S1 File (PDF)

S1 Data (CSV)

S1 Appendix (PDF)
