# Supplementary material for: An AI-based intervention for improving undergraduate STEM learning
Source: PLoS One. 2023 Jul 19;18(7):e0288844. doi: 10.1371/journal.pone.0288844 (PMC10355461; doi:10.1371/journal.pone.0288844)
Supplement: S1 File — (PDF) [file pone.0288844.s001.pdf]

Default Report

2018-Fall-GradeForecastingAppSurvey

December 27, 2022 7:07 PM MST

Q1 - How many times have you used the grade forecasting app during the semester?

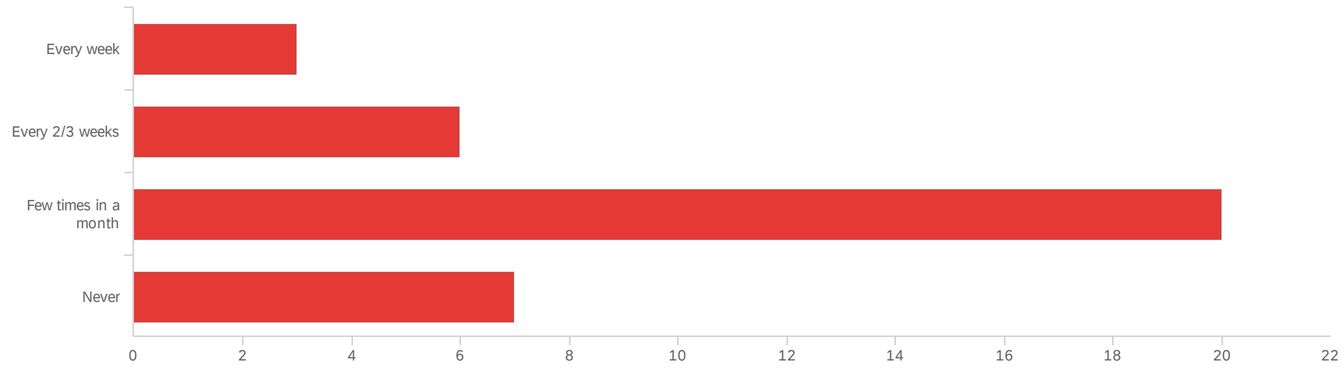

#	Field	Minimum	Maximum	Mean	Std Deviation	Variance	Count
1	How many times have you used the grade forecasting app during the semester?	1.00	4.00	2.86	0.82	0.68	36

#	Field	Choice Count	
1	Every week	8.33%	3
2	Every 2/3 weeks	16.67%	6
3	Few times in a month	55.56%	20
4	Never	19.44%	7
			36

Showing rows 1 - 5 of 5

Q2 - How useful were the predictions?

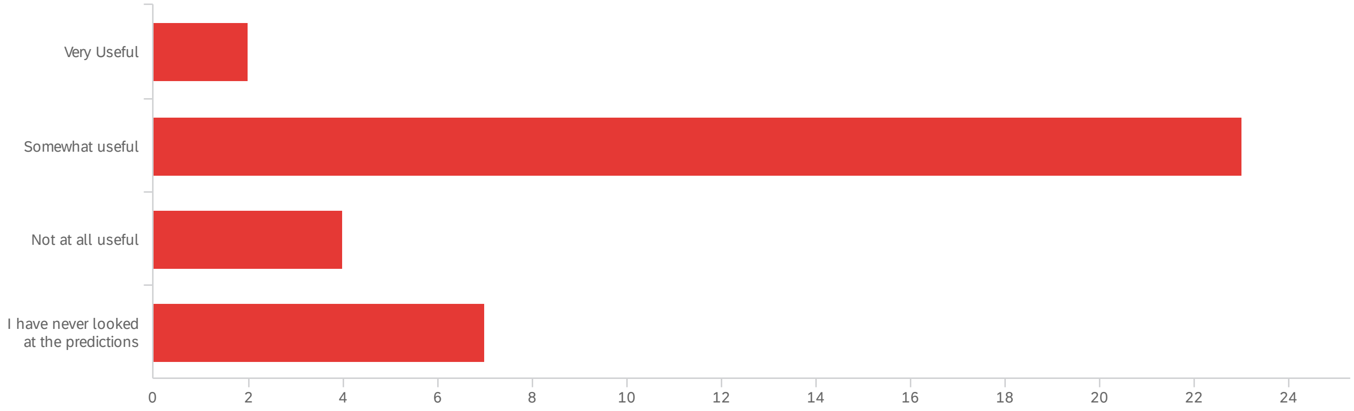

#	Field	Minimum	Maximum	Mean	Std Deviation	Variance	Count
1	How useful were the predictions?	1.00	4.00	2.44	0.86	0.75	36

#	Field	Choice Count	
1	Very Useful	5.56%	2
2	Somewhat useful	63.89%	23
3	Not at all useful	11.11%	4
4	I have never looked at the predictions	19.44%	7
			36

Showing rows 1 - 5 of 5

Q3 - Did you put more effort in your studies after seeing the predictions?

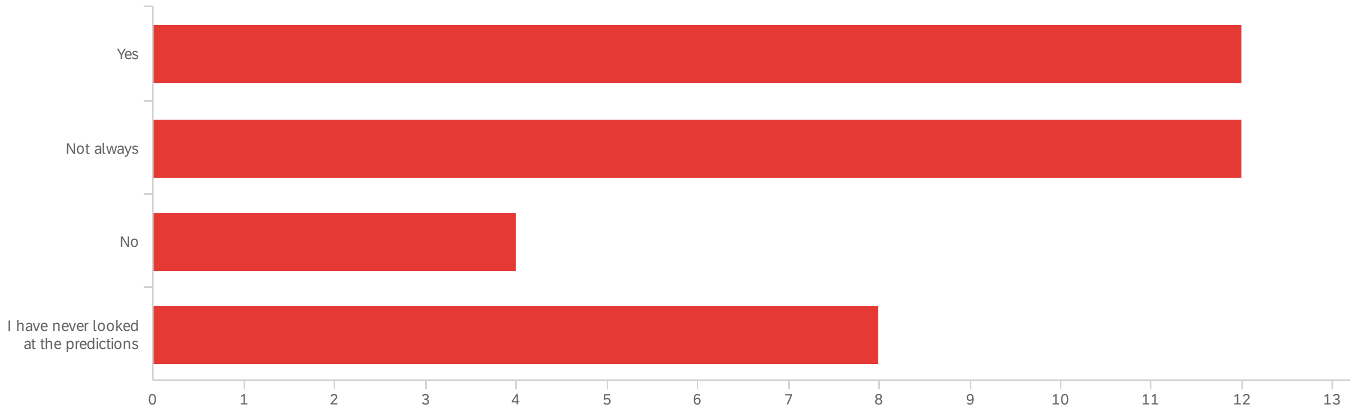

#	Field	Minimum	Maximum	Mean	Std Deviation	Variance	Count
1	Did you put more effort in your studies after seeing the predictions?	1.00	4.00	2.22	1.13	1.28	36

#	Field	Choice Count	
1	Yes	33.33%	12
2	Not always	33.33%	12
3	No	11.11%	4
4	I have never looked at the predictions	22.22%	8
			36

Showing rows 1 - 5 of 5

Q4 - Do you prefer to receive email notification of the predictions?

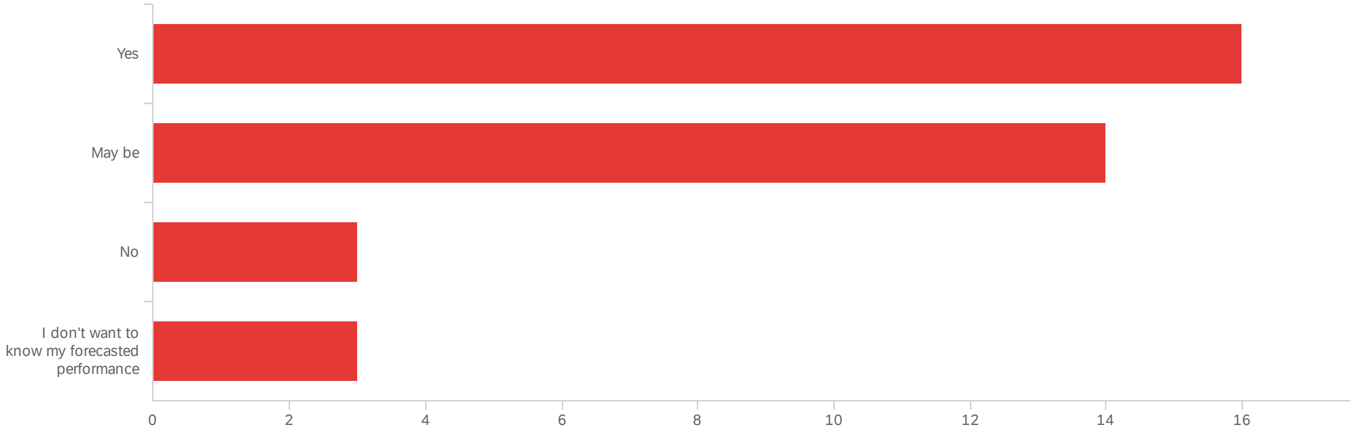

#	Field	Minimum	Maximum	Mean	Std Deviation	Variance	Count
1	Do you prefer to receive email notification of the predictions?	1.00	4.00	1.81	0.91	0.82	36

#	Field	Choice Count	
1	Yes	44.44%	16
2	May be	38.89%	14
3	No	8.33%	3
4	I don't want to know my forecasted performance	8.33%	3
			36

Showing rows 1 - 5 of 5

Q5 - Do you want to have a mobile version of the forecasting app?

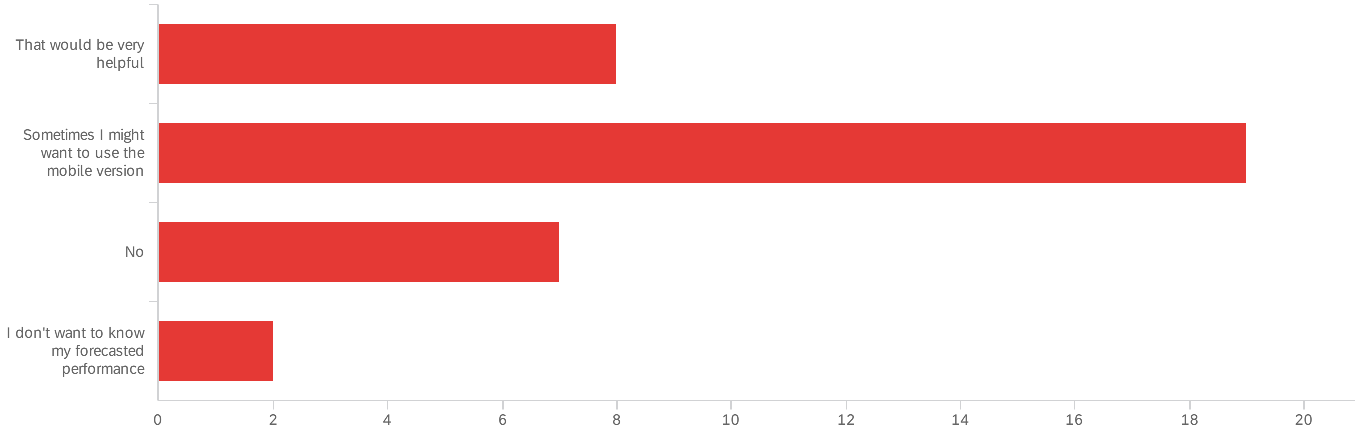

#	Field	Minimum	Maximum	Mean	Std Deviation	Variance	Count
1	Do you want to have a mobile version of the forecasting app?	1.00	4.00	2.08	0.79	0.63	36

#	Field	Choice Count	
1	That would be very helpful	22.22%	8
2	Sometimes I might want to use the mobile version	52.78%	19
3	No	19.44%	7
4	I don't want to know my forecasted performance	5.56%	2
			36

Showing rows 1 - 5 of 5

Q6 - Do you want to have a similar grade forecasting app for all courses?

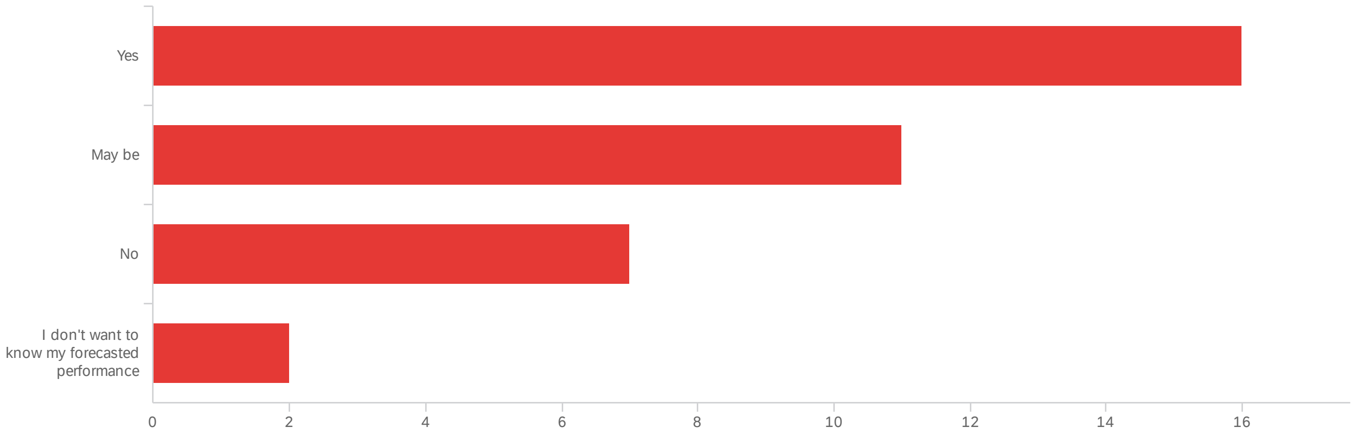

#	Field	Minimum	Maximum	Mean	Std Deviation	Variance	Count
1	Do you want to have a similar grade forecasting app for all courses?	1.00	4.00	1.86	0.92	0.84	36

#	Field	Choice Count	
1	Yes	44.44%	16
2	May be	30.56%	11
3	No	19.44%	7
4	I don't want to know my forecasted performance	5.56%	2
			36

Showing rows 1 - 5 of 5

Q7 - Have you interacted with your 3D avatar?

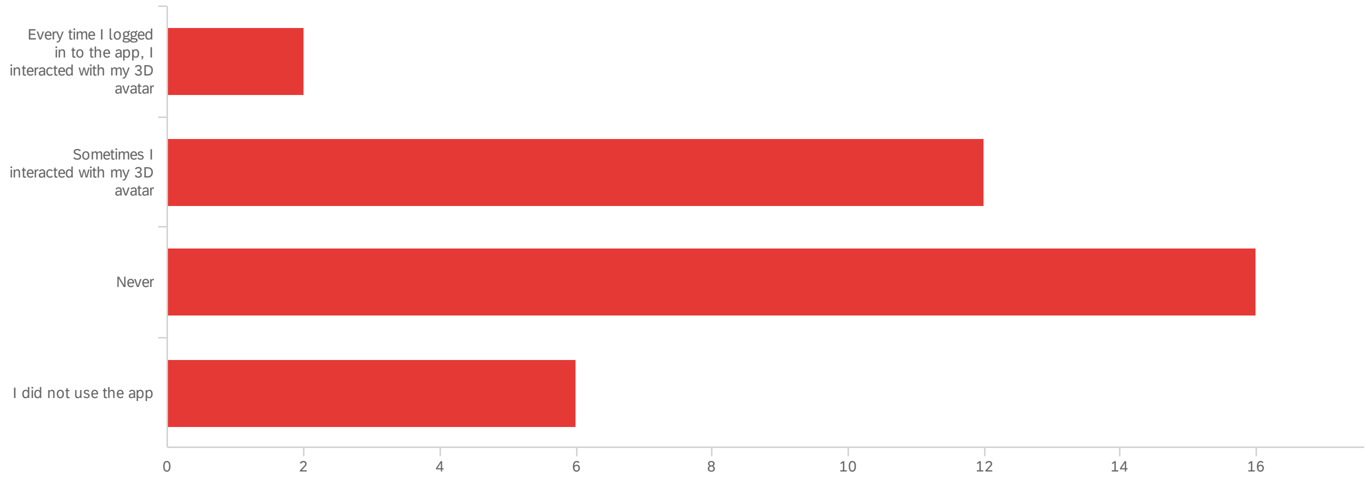

#	Field	Minimum	Maximum	Mean	Std Deviation	Variance	Count
1	Have you interacted with your 3D avatar?	1.00	4.00	2.72	0.80	0.65	36

#	Field	Choice Count	
1	Every time I logged in to the app, I interacted with my 3D avatar	5.56%	2
2	Sometimes I interacted with my 3D avatar	33.33%	12
3	Never	44.44%	16
4	I did not use the app	16.67%	6
			36

Showing rows 1 - 5 of 5

Q8 - Do you like the idea of your 3D avatar telling you about your future performance in the course?

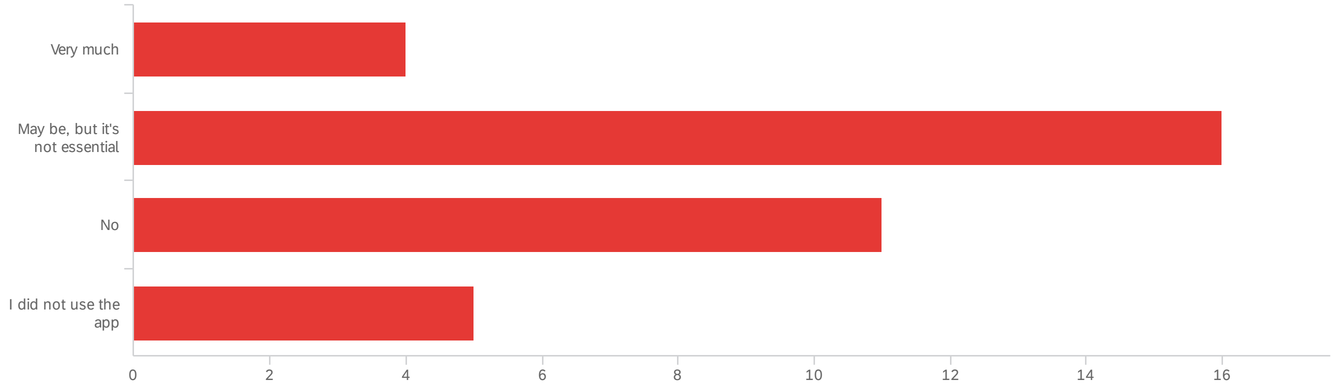

#	Field	Minimum	Maximum	Mean	Std Deviation	Variance	Count
1	Do you like the idea of your 3D avatar telling you about your future performance in the course?	1.00	4.00	2.47	0.87	0.75	36

#	Field		Choice Count
1	Very much	11.11%	4
2	May be, but it's not essential	44.44%	16
3	No	30.56%	11
4	I did not use the app	13.89%	5
			36

Showing rows 1 - 5 of 5

Q9 - How would you evaluate the overall design of the app interface?

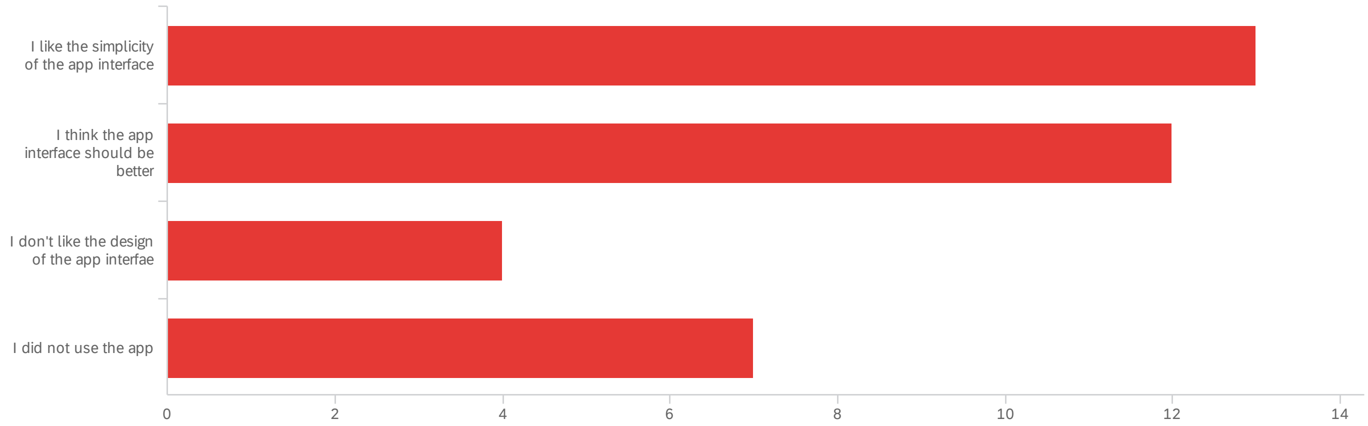

#	Field	Minimum	Maximum	Mean	Std Deviation	Variance	Count
1	How would you evaluate the overall design of the app interface?	1.00	4.00	2.14	1.11	1.23	36

#	Field	Choice Count	
1	I like the simplicity of the app interface	36.11%	13
2	I think the app interface should be better	33.33%	12
3	I don't like the design of the app interfae	11.11%	4
4	I did not use the app	19.44%	7
			36

Showing rows 1 - 5 of 5

Q10 - Do you want to be a part of the developer's team to work on the improvement of this app?

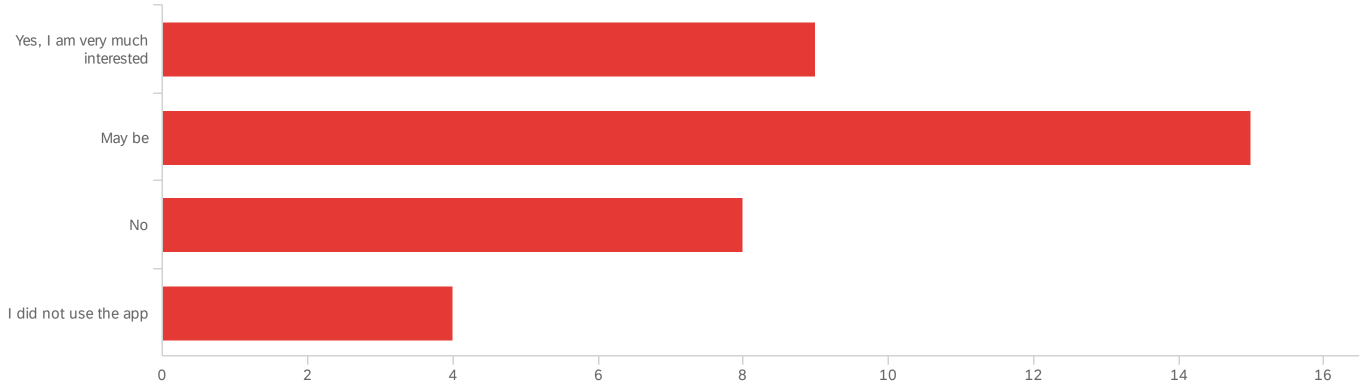

#	Field	Minimum	Maximum	Mean	Std Deviation	Variance	Count
1	Do you want to be a part of the developer's team to work on the improvement of this app?	1.00	4.00	2.19	0.94	0.88	36

#	Field		Choice Count
1	Yes, I am very much interested	25.00%	9
2	May be	41.67%	15
3	No	22.22%	8
4	I did not use the app	11.11%	4
			36

Showing rows 1 - 5 of 5

End of Report
