# Supplementary material for: An AI-based intervention for improving undergraduate STEM learning
Source: PLoS One. 2023 Jul 19;18(7):e0288844. doi: 10.1371/journal.pone.0288844 (PMC10355461; doi:10.1371/journal.pone.0288844)
Supplement: S1 Appendix — (PDF) [file pone.0288844.s003.pdf]

Supporting information

Performance of the Predictive ML Models. We used 80% of the N=300+ students' data to train the ML-based framework and tested it using 20% of the data. The performance of our predictive machine learning models is given in Table 5. The model used more features for the later predictions, as a result, the quality of the predictions improved. The model was tuned to increase precision and recall for the "You are At-Risk" group [1]. However, this improvement came at the cost of lower precision and recall for the "You are Prone-to-Risk" group.
Python code for Barnard's test.

```
import scipy.stats as stats
res = stats.barnard_exact([[3, 9], [29, 24]],
                          alternative="less", pooled=False)
print(res.statistic)
print(res.pvalue)
```

Table 5. Performance of the machine learning models.

		Predictions by		
**Class**	**Measure**	**$\mathcal{M}_6$ at 6 wks**	**$\mathcal{M}_9$ at 9 wks**	**$\mathcal{M}_{12}$ at 12 wks**
At-Risk	Precision	0.70	0.79	0.92
	Recall	0.79	0.90	0.83
	F1	0.74	0.84	0.87
Prone-To-Risk	Precision	0.44	0.58	0.62
	Recall	0.38	0.52	0.76
	F1	0.41	0.55	0.68
Ok	Precision	0.68	0.74	0.81
	Recall	0.56	0.59	0.74
	F1	0.61	0.66	0.77
Good	Precision	0.66	0.76	0.84
	Recall	0.79	0.92	0.88
	F1	0.72	0.83	0.86
Overall Accuracy		0.64	0.73	0.80

References

1. Hasan MR, Aly M. Get More From Less: A Hybrid Machine Learning Framework for Improving Early Predictions in STEM Education. In: The 6th Annual Conf. on Computational Science and Computational Intelligence, CSCI 2019 (CSCI'19); 2019.
